# Ceanothus Americanus

**Published:** 1868

**Authors:** D. L. Phares

**Affiliations:** Newtonia, Miss.


Medical and Surgical Journal.

NEW SERIES.

VOL. IXA. JULY AND AUGUST, 1868. No. 3.

### ORIGINAL COMMUNICATIONS.

### ARTICLE I.

*Ceanothus Americanus.* (*Red Root, New Jersey Tea.*) By D. L. PHARES, M. D., Newtonia, Miss.

The natural order Rhamnaceæ contains several genera of valuable medicinal plants indigenous to the Southern States: as Ceanothus, Rhamnus, Frangula, Berchemia, &c.

In this paper, I will limit myself to a brief notice of the first, which is found in most of the Southern States, perhaps in all; growing in dry woods, preferring, so far as I have observed, gentle slopes, and a soil with a considerable per-percentage of sand. Calyx 5—cleft with the tubes aduate to the ovary and persistent, the lobes connivent deciduous. Petals 5, longer than the calyx, hooded, long-clawed, stamens exserted. Style 3—parted; the small flowers and pedicles white; drupe dry, composed of three 2-valved, 1-seeded nullets; plant suffrutescent, 1 to 2 feet high, consisting of a few to many small stems, rising from one large root, of a dark red color, and bark containing many small white veins, and having the odor of peach leaves; branches light-green, pubescent; leaves deciduous, petioled, 3-ribbed, more or less pubescent, often nearly smooth, three-quarters to three inches long, ovate or ovate,—lanceolate, acute or obtuse, sharply serrate. Other varieties or species with leaves only iwo to five lines long.

The dried leaves, slightly bitter and astringent, have the odor of the black tea of commerce, as a substitute for which they acquired cousiderable notoriety during the war of American Independence. The leaves, flowers, and root have been used medicinally, and reputed astringent, diuretic, tonic, expectorant, sedative, antispasmodic, antisyphilitic, &c. The Indians used it in fevers, dysentery, epilepsy, asthma, chronic bronchitis, pertussis, gonorrhœa, syphilis, &c. An Indian remedy, for dysentery, of considerable reputation, the formula of which has never been made public, is prepared by boiling the root of the plant, cut in small pieces, in the crushed dupes of the Callicorpa; to which add sugar and molasses, and boil to rich syrup. Dose f. ℥ ss.—i. p. r. n. I have tried this remedy in a few cases; not enough to test it fairly. Not finding its action so prompt and satisfactory as other remedies, I did not experiment much with it. This preparation, and others, have been long used, and are still used, in gonorrhœa and syphilis, with an alleged success, which, if true, surpasses that of any other remedy within my knowledge, curing inveterate cases of the latter in a fortnight, and the former in three days. Having no experience with it in the premises, I am not prepared to admit the truth of these extraordinary claims.

In the Boston Medical and Surgical Journal, 1835, Dr. Hubbard commends Ceanothus, in decoction, for dysentery and ulcerated sore throat of scarlatina; and, in aphthæ of children and sore mouth after fevers, he succeeded with it when all other remedies failed. Other authorities, American and European, might be cited; but I presume enough has been said on the use of this plant in the diseases mentioned to excite attention; for if it possess only in part the properties ascribed to it, it is certainly worthy of investigation.

My object, however, in writing, is from my own knowledge and experience to add to the already long list of valuable properties ascribed to this plant, one more, which, so far as I know, has escaped the notice of others. During the late civil war I commenced using it for splenitis; and so well satisfied have I been with the results, that for six years I do not remember using anything else for enlarged spleen.

In this affection, I have never found any remedy superior to Ceanothus. I have used it in the worst cases I ever saw, from tender infancy to advanced age. (Two of the largest spleens, relatively, I ever saw, were in the same family, one a child a few months old, and the next year one a few weeks old, probably born with enlarged spleen.) I have yet to see or hear of its failure in a single case, however inveterate.

During the war, I had neither time nor means to procure elegant preparations, so I ordered a bottle loosely filled with the chipped root, carefully putting in all the bark, and then filled with spirit. frumenta. It was ready for use in a week, and the dose for an adult, f. ℥ ss.—i ter. die.

I directed the same tincture rubbed on the surface over the spleen, bis. die., the patient having the side turned towards and near a fire. A second bottle is seldom required. In chronic cases, when the organ is no longer tender, under the use of this tincture, even without the friction, it soon becomes painful and tender, and then sinks very rapidly to its normal size, and so remains, the patient being no longer conscious of its presence. I have not seen a relapse occur in any case; though no doubt such would be likely to occur under circumstances favoring it.

We have other indigenous remedies for this affection, which I have tried and found very valuable, of which, at another time, I may have something to say.

